# Tomato Polyphenols Protect Human Red Blood Cells from Mercury-Induced Oxidative Stress and Phosphatidylserine Exposure

**DOI:** 10.3390/cimb48060567

**Published:** 2026-05-28

**Authors:** Claudia Moriello, Nicola Alessio, Rosario Finamore, Chiara De Rosa, Chiara Schiraldi, Caterina Manna, Stefania D’Angelo, Pasquale Perrone

**Affiliations:** 1 Department of Experimental Medicine, University of Campania “Luigi Vanvitelli”, 80138 Naples, Italy; claudia.moriello@unicampania.it (C.M.); nicola.alessio@unicampania.it (N.A.); rosario.finamore@unicampania.it (R.F.); chiara.schiraldi@unicampania.it (C.S.); 2 School of Medicine and Surgery, Department of Clinical Medicine, Surger University of Naples Federico II, 80131 Naples, Italy; chiara.derosa8@studenti.unina.it; 3 Department of Precision Medicine, School of Medicine, University of Campania “Luigi Vanvitelli”, 80138 Naples, Italy; caterina.manna@unicampania.it; 4 Department of Medical, Movement, and Wellbeing Sciences, Parthenope University of Naples, 80133 Naples, Italy; 5 Department of Psychology and Health Sciences, Pegaso Telematic University, 80143 Naples, Italy

**Keywords:** cardiovascular risk, mercury toxicity, oxidative stress, phosphatidylserine externalization, reactive oxygen species, red blood cells, tomato polyphenols

## Abstract

Oxidative stress plays a central role in the development of cardiovascular diseases (CVDs) and is strongly influenced by environmental toxicants such as heavy metals. Mercury (Hg) is a well-known pro-oxidant agent capable of disrupting cellular redox homeostasis, promoting reactive oxygen species (ROS) generation, and inducing structural and functional alterations in circulating blood cells. In particular, erythrocytes (RBC) are highly susceptible to oxidative damage due to their continuous exposure to oxygen and limited antioxidant repair mechanisms. In this study, we investigated the protective effects of polyphenolic extracts obtained from two tomato (*Solanum lycopersicum*) cultivars, Piccadilly and Piennolo, against HgCl_2_-induced oxidative stress in human RBC. Chemical characterization revealed that Piennolo extracts contained higher levels of total polyphenols, flavonoids, and *ortho*-diphenols compared with Piccadilly samples. Despite these differences, both extracts showed comparable antioxidant capacity. In RBC exposed to HgCl_2_, tomato extracts significantly reduced ROS production, lipid peroxidation (TBARS), methemoglobin formation, and sulfhydryl depletion, while restoring intracellular glutathione levels. Importantly, pretreatment with the extracts markedly decreased phosphatidylserine externalization, indicating preservation of membrane phospholipid asymmetry and a reduction in the procoagulant phenotype induced by oxidative stress. Overall, these findings suggest that bioactive compounds present in polyphenol-rich tomato extracts exert protective effects on RBC through antioxidant and membrane-stabilizing mechanisms.

## 1. Introduction

Cardiovascular diseases (CVDs) remain the leading cause of morbidity and mortality worldwide, representing one of the major public health challenges in both industrialized and developing countries. Their pathogenesis and progression are multifactorial, arising from the complex interplay of genetic, metabolic, inflammatory, and environmental factors [1,2]. In addition to traditional risk factors, such as dyslipidemia, diabetes, smoking, and physical inactivity, chronic exposure to environmental contaminants, particularly heavy metals, is now widely recognized as an emerging determinant of cardiovascular risk [3,4]. Among these, mercury (Hg) stands out because of its high chemical reactivity, bioaccumulative properties, and ability to interfere with multiple biological systems, making it a significant cardiotoxic agent [5,6].

Hg exposure, which occurs mainly through diet, environmental pollution, and certain industrial activities, has been associated with endothelial dysfunction, coagulation abnormalities, chronic inflammation, and increased systemic oxidative stress [7,8]. At the cellular level, Hg exerts its toxicity by binding to protein sulfhydryl groups (SH), inhibiting key enzymes of redox metabolism, and impairing intracellular antioxidant systems, such as glutathione peroxidase and catalase [9]. This imbalance promotes the accumulation of reactive oxygen species (ROS), which damage lipids, proteins, and nucleic acids, thereby creating a strongly pro-oxidant environment closely linked to CVD pathogenesis [5].

Human erythrocytes (RBCs), traditionally regarded as simple oxygen carriers, are now recognized as active players in cardiovascular pathophysiology [10]. Owing to their high iron content and continuous exposure to molecular oxygen, RBCs are particularly vulnerable to oxidative damage [11]. Moreover, RBCs represent a major biological target of Hg toxicity. By interacting with membrane proteins and hemoglobin, Hg amplifies ROS generation, inducing lipid peroxidation, membrane stiffening, and cytoskeletal dysfunction, which in turn compromises RBC deformability and survival [12].

Among the phenomena triggered by Hg-induced oxidative stress, a particularly relevant mechanism in the cardiovascular context is the externalization of phosphatidylserine (PS) on the RBC membrane surface. In healthy cells, PS is normally confined to the cytoplasmic leaflet of the plasma membrane, maintaining a phospholipid asymmetry that is crucial for cellular homeostasis [13]. However, under conditions of severe oxidative stress and increased intracellular calcium (Ca^2+^), PS can translocate to the outer leaflet, a process known as PS externalization [14,15]. This event acquires major pathophysiological significance in CVDs, as externalized PS acts as a procoagulant signal that actively promotes thrombogenesis and adhesion to endothelial cells and platelets, thereby contributing to a prothrombotic state that favors the development of acute cardiovascular events [16,17].

Within this pathophysiological framework, the identification of strategies capable of protecting RBC from oxidative stress and preserving membrane asymmetry represents a highly relevant clinical objective. Polyphenols, a large and structurally heterogeneous class of bioactive plant-derived compounds, are well known for their antioxidant properties [18]. Beyond their radical-scavenging activity, polyphenols can stabilize biological membranes, chelate metal ions, and modulate signaling pathways involved in oxidative stress and cell death, suggesting a potential role in RBC protection and in the prevention of thrombotic events [19]. A growing body of literature has extensively characterized the pleiotropic mechanisms underlying the biological activity of polyphenols. In addition to their direct antioxidant capacity, these compounds have been shown to regulate redox-sensitive transcription factors (e.g., Nrf2 and NF-κB), modulate intracellular signaling cascades involved in inflammation and apoptosis, and influence ion transport and membrane fluidity [20,21,22].

Tomato (*Solanum lycopersicum*) is one of the most extensively studied foods in the context of cardiovascular health, mainly due to its content of lycopene, a carotenoid with strong antioxidant and anti-inflammatory properties [23]. However, tomato is also a significant source of polyphenols, including flavonoids, phenolic acids, and quercetin derivatives, whose contribution to the cardiovascular benefits of tomato remains only partially explored.

In light of these considerations, the present study aimed to evaluate the protective effects of polyphenols extracted from two widely consumed tomato cultivars, Piccadilly (Sicily, Italy) and Piennolo (Campania, Italy), on human RBCs exposed to high concentrations of HgCl_2_. To characterize the phenolic profile of the extracts, total polyphenol, flavonoid, and ortho-diphenol contents were determined. In addition, several markers of oxidative stress, including thiobarbituric acid reactive substances (TBARS) and ROS formation, intracellular glutathione (GSH) levels, SH groups, and methemoglobin (MetHB), were assessed to evaluate the ability of the extracts to attenuate Hg-induced oxidative stress. Finally, PS exposure was analyzed to determine whether the extracts preserve membrane phospholipid asymmetry and reduce the prothrombotic properties of RBCs, thereby providing new evidence for the potential role of tomato polyphenols as nutraceutical agents in the prevention of cardiovascular complications associated with heavy-metal exposure.

## 2. Materials and Methods

### 2.1. Chemicals and Solutions

DCFH-DA (2′,7′-dichlorodihydrofluorescein diacetate), DTNB (5,5-dithiobis (2-nitrobenzoic acid) or Ellman’s reagent, PBS (phosphate-buffered saline), EDTA, TCA, TBA and HgCl_2_ were purchased from Sigma Chemical Co. (St. Louis, MO, USA). The AnnexinV-fluorescein isothiocyanate (V-FITC) apoptosis detection kit (556547, BD Pharmigen, Franklin Lakes, NJ, USA) was purchased from www.antibodies-online.com (Limerick, PA, USA). Methanol was obtained from Romil Ltd. (Cambridge, UK). The ABNOKA1622 assay kit was obtained from ABNOVA (Taipei, Taiwan). The MAK509 kit was obtained from Sigma-Aldrich (Burlington, MA, USA).

### 2.2. Polyphenol Extraction

A Tefal Rondo 500 homogenizer was used to homogenize 40 g of tomato samples using 40 mL of 80% methanol, 20% water, and 0.18 N HCl (15 mL of 12 N of HCl/L) for 5 min. After centrifugation (18,000× *g* for 25 min), the slurry was dried under vacuum using the Eppendorf Concentrator Plus. The dried extracts were dissolved in 10 mL of PBS and frozen at −80 °C until use [24]. Although the extraction procedure employed is widely used and optimized for the efficient recovery of polyphenols from plant matrices, it does not ensure absolute selectivity. Therefore, while the extracts are expected to be highly enriched in phenolic compounds, the presence of trace amounts of other co-extracted molecules cannot be completely excluded. However, given their likely negligible concentrations, it is reasonable to assume that the observed biological effects are largely driven by the polyphenol-rich fraction, although a contribution from trace co-extracted compounds cannot be completely excluded.

Furthermore, the acidified extraction medium was used to inhibit polyphenol oxidase activity and maximize phenolic recovery. Although acidic conditions may induce limited chemical modifications, their impact on the overall polyphenolic profile is considered minimal; however, this aspect cannot be completely excluded.

### 2.3. Polyphenolic, Flavonoids and Ortho-Diphenolic Contents

Folin–Ciocalteu phenolic reagent was used to assess the total polyphenolic content of tomato extracts. In particular, Folin–Ciocalteu phenolic reagent (0.5 mL), deionized water (0.9 mL), and Na_2_CO_3_ (7.5% *w*/*v*, 4 mL) were combined with the extracts (100 μL). A UV-3100PC spectrophotometer was used to detect the absorbance at 765 nm 2 h after incubation at room temperature. The measurement was compared to a standard curve of prepared catechin solutions and expressed in milligrams of catechin (CAEq) equivalent per 100 g FW (fresh weight) of tomato.

The AlCl_3_ colorimetric method was used to determine the total flavonoid concentration. In short, 120 μL of aqueous sodium acetate solution, 80 μL of 2% AlCl_3_ solution, and 80 μL of the sample extract were combined. The absorbance at 440 nm was measured after the reaction mixture was left in the dark at 25 °C for 2.5 h. The measurement was compared to a standard curve of prepared quercetin solutions and expressed as milligrams of quercetin equivalent per 100 g ± SD of tomato.

The Arnow reagent was used to determine the *o*-diphenolic concentration. In short, three solutions were added to the samples (400 μL) in the following order: solution A (0.5 M HCl, 400 μL), solution B (1.45 M NaNO_2_–0.4 M Na_2_MoO_4_, 400 μL), and solution C (1 M NaOH, 400 μL). The ortho-diphenolic content was determined by measuring the sample’s absorbance at 500 nm and comparing it to a reference curve of prepared catechin solutions. The results were represented as milligrams of CAE equivalent per 100 g ± SD tomato component [25].

### 2.4. High-Performance Liquid Chromatography (HPLC)

Analysis of the extracts was performed using an Ultimate 3000+ UPLC system (Thermo Fisher Scientific, Milan, Italy), equipped with Chromeleon software, version 7.4 (Thermo Fisher Scientific, Milan, Italy). Standard solutions were prepared in methanol (2.0–0.0625 µg/µL) and injected onto a reversed-phase column (EC250/4.6 NUCLEODUR 100-5 C18ec, Macherey-Nagel; distributed by Del Chimica, Naples, Italy).

The mobile phases consisted of (A) 0.1% formic acid in water, and (B) 0.1% formic acid in methanol. Elution was carried out using a step gradient of solvent B as follows: 0.0 min, 5.0% B; 5.0 min, 5.0% B; 55.0 min, 95.0% B; 60.0 min, 95.0% B; 65.0 min, 5.0% B; 70.0 min, 5.0% B. The flow rate was set at 0.7 mL/min, and the column temperature at 35 °C. Chromatograms were recorded by monitoring the absorbance at 280, 310, 325, and 360 nm.

### 2.5. Determination of Antioxidant Capacity

#### 2.5.1. Total Antioxidant Capacity Assay

The ABNOKA1622 assay kit was used to determine the tomato extract samples’ total antioxidant capacity (TAC). The procedure followed the manufacturer’s instructions. In short, 100 μL of the working solution, and 20 μL of tomato extract samples were combined in each well of a 96-well microplate. After 10 min of room temperature incubation, the absorbance at 570 nm was measured using an IMARK microplate reader. A Trolox-generated standard curve was used to assess TAC values.

#### 2.5.2. FRAP

The ferric-reducing antioxidant power (FRAP) assay was assessed using the MAK509 kit. The assay was carried out in accordance with the manufacturer’s suggested procedure. In short, 200 μL of working solution and 50 μL of tomato extract samples were combined in each well of a 96-well microplate. After 40 min of room temperature incubation, the absorbance at 590 nm was measured using an IMARK microplate reader. FRAP values were calculated using the following formula:

$$[FRAP](\mu M\;{Fe}^{3+}reduction\;potential)=\frac{Rsample-Rblank}{Slope}$$

where Rsample is the absorbance of samples, and Rblank is the absorbance of blank.

### 2.6. Preparation of Red Blood Cells and Treatment with HgCl_2_

Whole blood was obtained with informed consent from healthy volunteers at the University of Campania ‘Luigi Vanvitelli’ (Naples, Italy). It was gathered in heparinized tubes and centrifuged at 2000× *g* for 10 min at 4 °C. After removing the buffy coat, the RBC fraction was resuspended in Krebs solution, as described by Perrone et al. [24]. After pretreating RBC with several doses of tomato extracts (5,10 and 20 μM) for 15 min, they were incubated with HgCl_2_ (20 μM) for 4 h at 37 °C.

### 2.7. Total Sulfhydryl Group Content

Measurement of the total -SH groups was performed according to the method of Perrone et al. [12]. After centrifuging RBCs (35% hematocrit) at 1200× *g* for 5 min, a 100 μL sample was hemolyzed in 1 mL of distilled water. One mL of phosphate-buffered saline (PBS, pH 7.4) containing 1 mg of EDTA was mixed with 50 μL of the hemolysate. Samples were incubated for 30 min at 25 °C in the dark after 30 μL of 10 mM 5,5′-dithiobis-(2-nitrobenzoic acid) (DTNB) was added to initiate the reaction. In parallel, control samples devoid of DTNB or cell lysate were processed. The UV-3100PC spectrophotometer was used to detect absorbance at 412 nm following incubation. Data were normalized to total protein content and results are expressed as μM TNB/mg protein.

### 2.8. Determination of MetHb Levels

With a few adjustments, MetHb levels were measured according to the method of Naoum et al. [26]. The assay is based on spectrophotometric measurements of the differential absorbance of oxyhemoglobin at 540 nm and MetHb at 630 nm.

In 1975 μL of hypotonic buffer (2.5 mM NaH_2_PO_4_, pH 7.4, at 4 °C), a 25 μL sample of RBC at 40% hematocrit was lysed. Cell membranes were then extracted from the lysates by centrifuging them at 13,000× *g* for 15 min at 4 °C. The UV-3100PC spectrophotometer (West Chester, PA, USA) was used to test the supernatant’s absorbance.

MetHb levels were expressed as a percentage of total hemoglobin using the following equation:

% MetHb = (OD_630_/OD_540_) × 100,

where OD represents optical density.

### 2.9. TBARS Level Measurement

With a few small modifications, TBARS levels were tested in accordance with Mendanha et al. [27]. Following incubation, samples were centrifuged (1200× *g*, 5 min) and resuspended in isotonic solution. RBCs were suspended at 20% hematocrit. After adding 10% (*w*/*v*) trichloroacetic acid (TCA) to a 1.5 mL aliquot of RBC solution, it was centrifuged at 3000× *g* for 10 min. The mixture was then incubated at 95 °C for 30 min after 1 mL of 1% TBA solution (made in hot distilled water) was added to the supernatant. Using a UV-3100PC spectrophotometer, TBARS levels were measured spectrophotometrically by deducting 20% of the absorbance at 453 nm from the absorbance at 532 nm. Results are expressed as μM TBARS, using a molar extinction coefficient of 1.56 × 10^5^ M^−1^·cm^−1^.

### 2.10. ROS Determination

The DCF assay, as outlined by Notariale et al. [10], was used to measure ROS production. In short, 10 μM 2′,7′-dichlorodihydrofluorescein diacetate (DCFH-DA) was incubated with 250 μL of intact RBCs at 10% hematocrit for 15 min at 37 °C. Following incubation, the cells were centrifuged at 1200× *g* for 5 min at room temperature. After that, RBCs were treated for 1 h in the dark. After different treatment durations (15, 30, 45, and 60 min), the fluorescence intensity of the oxidized product dichlorofluorescein (DCF) was measured at 502 nm excitation and 520 nm emission using 20 μL of RBC suspension diluted in 2 mL of distilled water. ROS levels were expressed as fluorescence intensity per mg of hemoglobin. Fluorescence measurements were performed using a UV-3100PC spectrophotometer.

### 2.11. GSH Assay

The amount of intracellular GSH was measured using a DTNB reaction. After incubation, 0.25 mL of RBC suspension was centrifuged for 5 min at 800× *g*. RBCs were lysed by adding 0.6 mL of cold distilled water after the supernatant was again removed. Then, 0.6 mL of a cold metaphosphoric acid solution was added to precipitate the proteins. To remove the precipitated proteins, the samples were centrifuged at 18,000× *g* for 10 min after being incubated at 4 °C for five minutes. A 0.45 mL aliquot of the resulting supernatant was mixed with an equal volume of 0.3 M Na_2_HPO_4_. To this mixture, 0.1 mL of DTNB solution (20 mg DTNB dissolved in 100 mL of water having 1% sodium citrate) was added. After 10 min of incubation at room temperature, absorbance was measured at 412 nm using a UV-3100PC spectrophotometer.

### 2.12. Detection of Phosphatidylserine Exposure

RBCs (hematocrit 0.4%) were suspended in 500 μL of 1× binding buffer with 1.5 μL of Annexin-V apoptosis detection reagent. They were then incubated in the dark for 45 min at room temperature. Fluorescence assessment was performed with BD AccuriC6, and data were analyzed on the FACS Calibur flow cytometer and evaluated with FlowJo V10 software.

### 2.13. Statistical Analyses

Data evaluations were expressed as the means ± S.D. of 3 independent experiments performed in triplicate with RBCs from different donors. The significance of differences was determined by one-way ANOVA followed by Tukey’s post hoc multiple comparisons test. GraphPad Prism 10 was utilized for statistical analysis.

## 3. Results

### 3.1. Polyphenolic, Flavonoids and Ortho-Diphenolic Contents

The analysis of total polyphenols, flavonoids, and *ortho*-diphenols in the extracts obtained from the two different tomato cultivars revealed that Piennolo tomatoes exhibited higher levels of all three classes of compounds.

As reported in Table 1, the total polyphenol content in Pien. extracts showed an increase of approximately 15% compared to Picc. samples. With regard to ortho-diphenol content, an increase of approximately 50% was observed in Pien. samples. Finally, the flavonoid content in Pien. extracts was approximately threefold higher than that detected in Picc. samples.

### 3.2. High-Performance Liquid Chromatography (HPLC)

**Figure 1:**
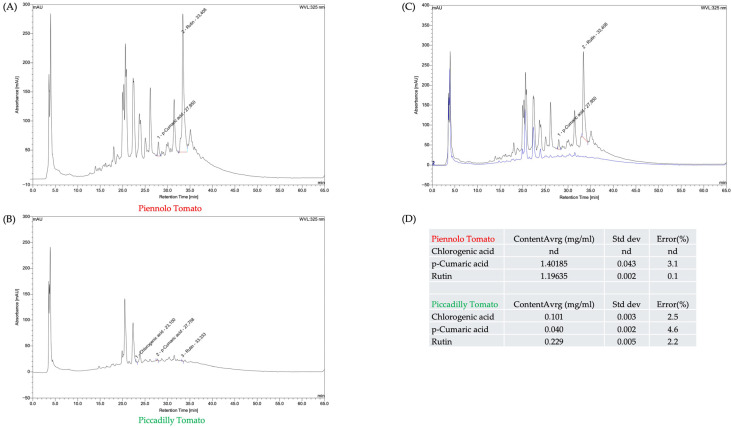
HPLC chromatograms of phenolic compounds in tomato samples recorded at 325 nm. (**A**) Chromatographic profile of Piennolo tomato; (**B**) chromatographic profile of Piccadilly tomato; (**C**) overlay of the two chromatograms highlighting differences in peak intensity and distribution; (**D**) quantitative determination of selected phenolic compounds (chlorogenic acid, p-coumaric acid, and rutin) expressed as mg/mL, including mean values, standard deviation, and percentage error. Retention times (Rt) are indicated for the identified compounds.

The HPLC chromatograms recorded at 325 nm reveal distinct phenolic profiles between the two tomato varieties analyzed. In the Pien. sample (Figure 1A), a highly complex chromatographic profile is observed, with numerous peaks distributed across the entire retention time range. In particular, p-coumaric acid (Rt ≈ 27.95 min) and rutin (Rt ≈ 34.08 min) are clearly identified, showing high signal intensities and suggesting relatively elevated concentrations of these compounds. In contrast, chlorogenic acid was not detected in this sample.

The chromatogram of the Picc. sample (Figure 1B) appears less complex and exhibits overall lower signal intensities. In this case, in addition to p-coumaric acid (Rt ≈ 27.76 min) and rutin (Rt ≈ 33.33 min), a peak attributable to chlorogenic acid (Rt ≈ 23.10 min) is also detected, although with relatively low intensity.

The direct comparison of chromatograms (Figure 1C) visually confirms the qualitative and quantitative differences between the two samples, highlighting a higher relative abundance of compounds detected at 325 nm in Pien. compared to Picc.

Quantitative data (Figure 1D) support these observations: in Pien., p-coumaric acid and rutin show mean concentrations of 1.40 mg/mL and 1.20 mg/mL, respectively, while chlorogenic acid was not detected. In Picc., all three compounds are present but at significantly lower concentrations (chlorogenic acid: 0.101 mg/mL; p-coumaric acid: 0.040 mg/mL; rutin: 0.229 mg/mL).

Overall, these results highlight marked differences in phenolic content between the two varieties, with Piennolo characterized by a richer and more concentrated profile, particularly in terms of p-coumaric acid and rutin.

### 3.3. Antioxidant Capacity

**Figure 2:**
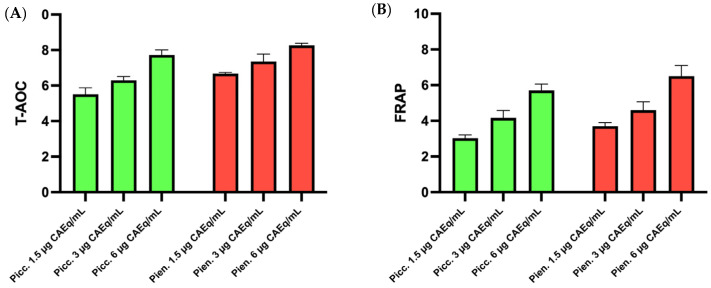
T-AOC assay of Picc. and Pien. tomato samples (**A**) and FRAP assay Picc. and Pien. tomato samples (**B**).

The first step in evaluating the cytoprotective potential of tomato extracts was the assessment of their antioxidant activity. As shown in Figure 2, both samples analyzed, Pien. and Picc., exhibited comparable antioxidant activity profiles, with no significant differences observed between the two extracts across the tested concentration range.

In both cases, antioxidant activity displayed a clear dose-dependent trend, characterized by a progressive increase with increasing concentration. Notably, a marked enhancement of antioxidant activity was observed at the highest concentration tested (20 μM), indicating a greater radical scavenging capacity under these experimental conditions.

The reliability of these findings is further supported by the use of two different antioxidant activity assays, which yielded consistent and overlapping results, thereby confirming the robustness of the antioxidant profiles observed for both extracts.

### 3.4. Total Sulfhydryl Group Content

SH groups, mainly present in cysteine residues of proteins and in low-molecular-weight thiols, such as GSH, play a crucial role in maintaining cellular redox balance and protecting cells against oxidative damage. Because they are highly susceptible to oxidation, variations in –SH group contents are widely used as sensitive biomarkers of oxidative stress and redox homeostasis disruption. Therefore, quantifying SH groups in RBCs represents a crucial parameter to monitor the toxic effects of oxidative agents, such as heavy metals, and to assess the protective potential of antioxidant compounds.

**Figure 3:**
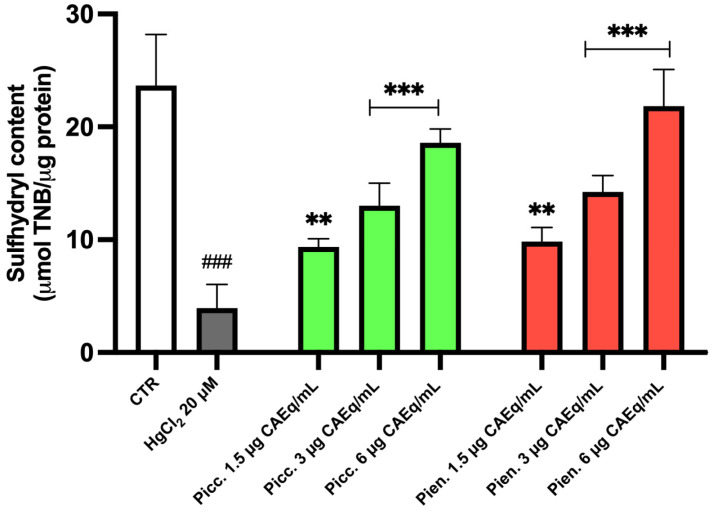
Effects of HgCl_2_ and different tomato extracts on RBC SH group content. Cells were treated with HgCl_2_ and increasing concentrations of Picc. and Pien extracts. Data are the means ± SD (*n* = 3). Statistical analysis was performed with one-way ANOVA followed by Tukey’s test. ### (*p* < 0.001) indicates a significant difference from CTR. *** (*p* < 0.001) and ** (*p* < 0.01) indicate a significant difference from HgCl_2_ treatment.

Figure 3 shows the quantification of SH groups in human RBCs exposed to HgCl_2_, either alone or following pretreatment with polyphenolic extracts from Picc. and Pien. tomatoes.

Exposure to HgCl_2_ resulted in a significant reduction in intracellular SH group content compared to untreated control cells, indicating a marked oxidative stress condition and impairment of cellular redox homeostasis.

Pretreatment of RBCs with tomato polyphenol-rich extracts exerted a protective effect against HgCl_2_-induced depletion of –SH groups, displaying a clear dose-dependent pattern. Specifically, increasing extract concentrations were associated with a progressive restoration of SH group levels relative to cells treated with HgCl_2_ alone, for both Picc. and Pien. extracts.

### 3.5. MetHb Levels

MetHb is an oxidized form of hemoglobin in which the iron atom is converted from the ferrous (Fe^2+^) to the ferric (Fe^3+^) state, resulting in a reduced ability to bind and transport oxygen. Under physiological conditions, MetHb is maintained at very low levels by efficient cellular reducing systems; however, oxidative stress, particularly that induced by heavy metals, can overwhelm these mechanisms, leading to MetHb accumulation [26]. For this reason, intracellular MetHb levels are widely used as a reliable biomarker of oxidative stress and hemoglobin oxidation in RBCs.

**Figure 4:**
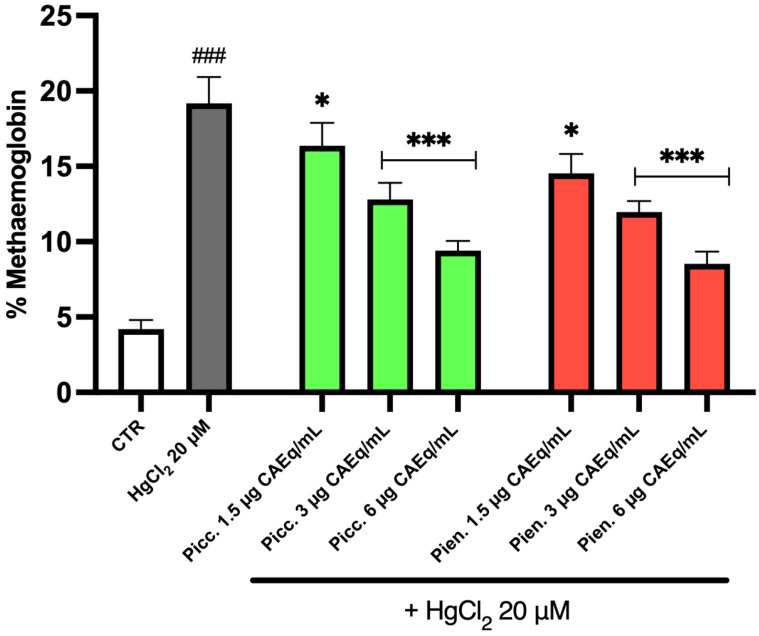
Effects of HgCl_2_ and different tomato extracts on RBC MetHb content. Cells were treated with HgCl_2_ and increasing concentrations of Picc. and Pien extracts. Data are the means ± SD (*n* = 3). Statistical analysis was performed with one-way ANOVA followed by Tukey’s test. ### (*p* < 0.001) indicates a significant difference from CTR. *** (*p* < 0.001) and * (*p* < 0.05) indicate a significant difference from HgCl_2_ treatment.

The analysis of intracellular MetHb levels highlighted the protective effect of tomato extracts against this specific marker of oxidative stress. In particular, the data shown in Figure 4 indicate that, as widely reported in the literature, exposure to HgCl_2_ induces a substantial increase, approximately fourfold, in intracellular MetHb levels compared to the control condition.

At the same time, the results clearly demonstrate that tomato extracts exert a remarkably strong protective effect. Indeed, at all three tested concentrations, a significant reduction in intracellular MetHb levels was observed compared to cells treated with the heavy metal alone. Notably, a dose-dependent trend was evident, with the highest concentration tested (20 μM) showing the most pronounced effect. It is also important to underline that, consistent with the other oxidative stress markers analyzed, the protective effects of Picc. and Pien. tomato extracts were highly comparable.

### 3.6. TBARS Levels

TBARS are widely used as an indirect index of lipid peroxidation, reflecting the extent of oxidative damage to cell membranes. The TBARS assay primarily detects MDA, a major end product of polyunsaturated fatty acid oxidation. Measurement of TBARS is particularly relevant in RBCs, as these cells are highly susceptible to oxidative stress due to their high oxygen exposure, abundance of polyunsaturated lipids in the membrane, and limited antioxidant repair capacity. Therefore, TBARS levels in RBCs represent a reliable marker for assessing oxidative damage and the protective effects of antioxidant compounds under pro-oxidant conditions [28].

**Figure 5:**
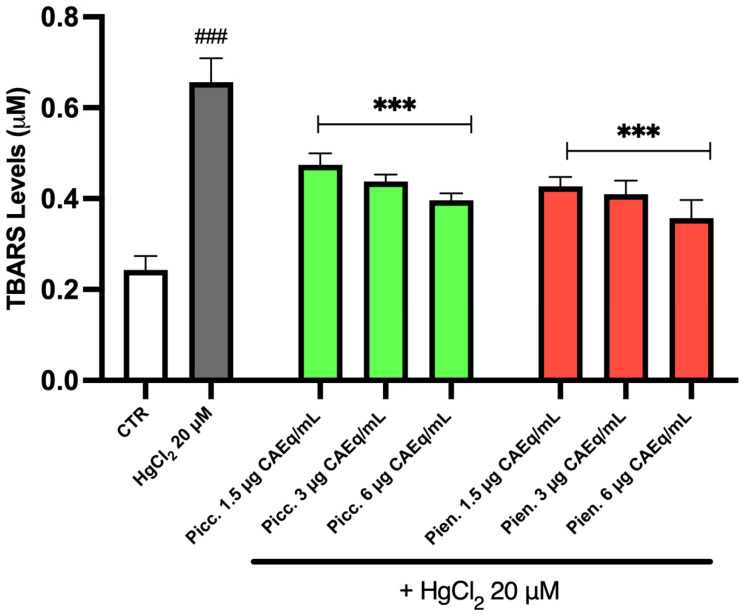
Effects of HgCl_2_ and different tomato extracts on RBC TBARS levels. Cells were treated with HgCl_2_ and increasing concentrations of Picc. and Pien extracts. Data are the means ± SD (*n* = 3). Statistical analysis was performed with one-way ANOVA followed by Tukey’s test. ### (*p* < 0.001) indicates a significant difference from CTR. *** (*p* < 0.001) indicates a significant difference from HgCl_2_ treatment.

The data reported in Figure 5 show a marked increase in TBARS levels in cells treated with the heavy metal, in agreement with previously published studies. Moreover, treatment with tomato extracts significantly reduced the extent of this alteration. In particular, a reduction slightly exceeding 30% was observed following treatment with both Picc. and Pien. extracts at all tested concentrations. Although a mild dose-dependent trend can be detected, the extracts exhibited a comparable protective effect across the concentration range examined. Consistently, the antioxidant activity of the two different polyphenolic extracts appears to largely overlap.

### 3.7. ROS Generation

**Figure 6:**
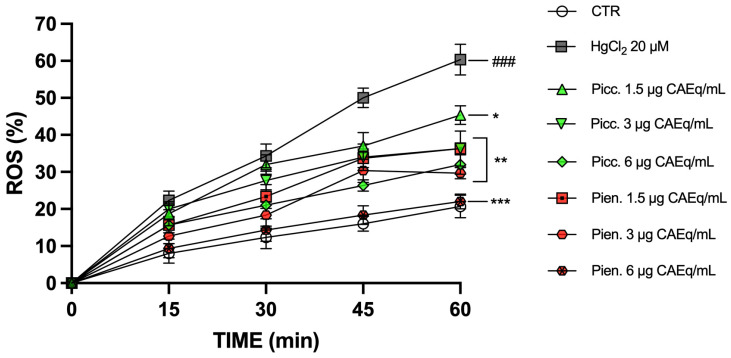
Effects of HgCl_2_ and different tomato extracts on ROS generation levels. Cells were treated with HgCl_2_ and increasing concentrations of Picc. and Pien extracts. Data are the means ± SD (*n* = 3). Statistical analysis was performed with one-way ANOVA followed by Tukey’s test. ### (*p* < 0.001) indicates a significant difference from CTR. *** (*p* < 0.001), ** (*p* < 0.01) and * (*p* < 0.05) indicate a significant difference from HgCl_2_ treatment.

Figure 6 shows the time-dependent trend (0–60 min) of ROS production. In the CTR group, a progressive and moderate increase in ROS levels was observed over time, reaching approximately 20% at 60 min. Treatment with HgCl_2_ 20 µM induced a significantly greater and time-dependent increase in ROS production compared to the control, with values exceeding 20% at 15 min, approximately 35% at 30 min, ~50% at 45 min, and reaching about 60–65% at 60 min.

Co-incubation with tomato extracts reduced the HgCl_2_-induced increase in ROS in a concentration-dependent manner. For both extracts, at 1.5 µg CAEq/mL ROS levels were lower than those observed in the HgCl_2_ group at all analyzed time points, reaching approximately 35–40% at 60 min. At 3 µg CAEq/mL, a further reduction was observed, with ROS values at 60 min around 30–35%. At 6 µg CAEq/mL, the decrease was more pronounced, with ROS levels at 60 min ranging between ~20% and ~25%, approaching control values.

When comparing the two extracts, at the same concentration and incubation times, Pien.-treated samples showed slightly lower ROS levels than the corresponding Picc.-treated samples, particularly at 3 and 6 µg CAEq/mL and at the longer time points (45–60 min), indicating a more marked reduction in ROS levels.

### 3.8. GSH Levels

GSH is the most abundant intracellular non-enzymatic antioxidant and plays a central role in maintaining redox homeostasis. In RBCs, which are continuously exposed to high oxygen tension and lack de novo protein synthesis, GSH represents a critical defense mechanism against oxidative damage. Therefore, intracellular GSH levels are widely used as a reliable biomarker to assess oxidative stress and cellular redox imbalance in RBC-based experimental models [29,30].

**Figure 7:**
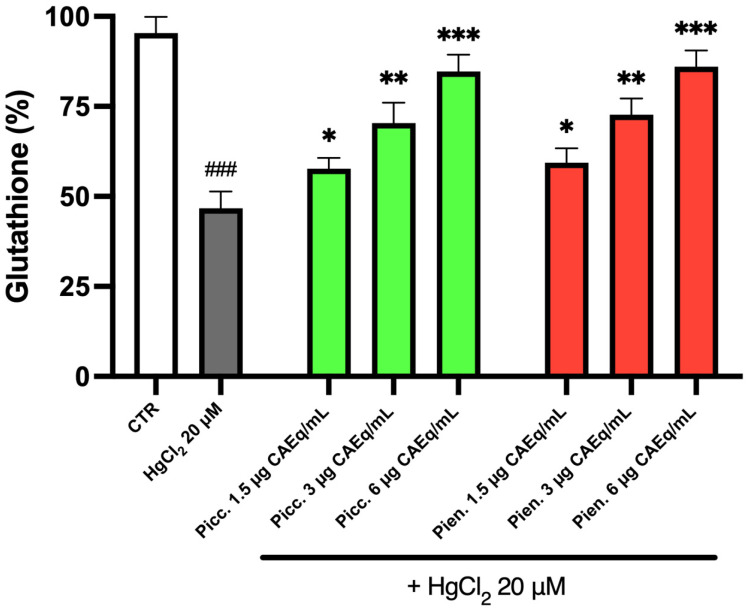
Effects of HgCl_2_ and different tomato extracts on GSH levels. Cells were treated with HgCl_2_ and increasing concentrations of Picc. and Pien extracts. Data are the means ± SD (*n* = 3). Statistical analysis was performed with one-way ANOVA, followed by Tukey’s test. ### (*p* < 0.001) shows a significant difference from CTR. *** (*p* < 0.001), ** (*p* < 0.01), and * (*p* < 0.05) indicate a significant difference from HgCl_2_ treatment.

Figure 7 shows intracellular GSH levels in RBC exposed to HgCl_2_ and pretreated with tomato extracts. Exposure to the heavy metal induced a marked depletion of intracellular GSH compared to untreated controls, with a reduction exceeding 50% of physiological levels. Pretreatment with tomato extracts significantly attenuated this toxic effect in a dose-dependent manner. In particular, at the highest tested concentration (6 µg CAEq/mL), GSH levels were restored to approximately 90% of basal values in both Picc. and Pien.-treated samples. Overall, these findings indicate that both tomato extracts exert a comparable protective effect against HgCl_2_-induced GSH depletion in RBCs.

### 3.9. Phosphatidylserine Exposure

**Figure 8:**
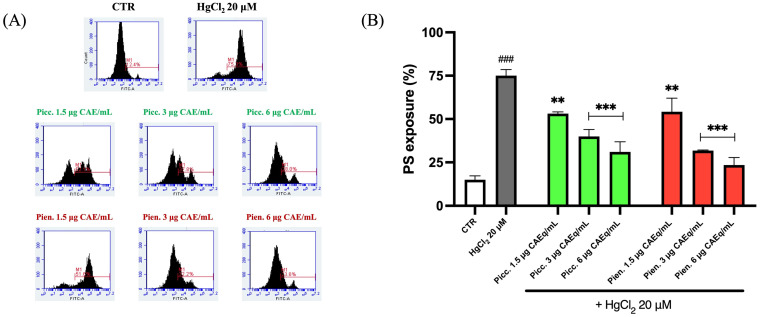
Effects of HgCl_2_ and different tomato extracts on PS exposure levels. (**A**) Representative image of flow cytometry data. (**B**) Histogram of the median data. Cells were treated with HgCl_2_ and increasing concentrations of Picc. and Pien extracts. Data are the means ± SD (*n* = 3). Statistical analysis was performed with one-way ANOVA followed by Tukey’s test. ### (*p* < 0.001) indicates a significant difference from CTR. *** (*p* < 0.001) and ** (*p* < 0.01) indicate a significant difference from HgCl_2_ treatment.

Figure 8 shows the analysis of PS externalization in RBCs, evaluated by flow cytometry. Data show that under basal conditions, control RBCs exhibited low PS exposure (~12–15%). In contrast, treatment with HgCl_2_ markedly increased PS externalization, reaching approximately 75%, indicating a strong toxic effect. Pretreatment with Picc. extract significantly attenuated this effect at all tested concentrations, with a progressive reduction observed from 1.5 to 6 µg CAEq/mL. A similar trend was observed for Pien., which also significantly decreased PS exposure in a dose-dependent manner.

## 4. Discussion

Oxidative stress is widely recognized as one of the central mechanisms involved in the onset and progression of numerous chronic diseases [31,32]. It arises from an imbalance between the production of ROS and the capacity of biological antioxidant defense systems to neutralize them. Under physiological conditions, ROS play important roles in cellular signaling and homeostasis. However, when their production exceeds the detoxifying capacity of endogenous antioxidant systems, ROS can damage cellular macromolecules such as lipids, proteins, and nucleic acids, ultimately leading to cellular dysfunction and tissue injury [33,34,35].

Among the various pathological conditions associated with oxidative stress, CVDs represent some of the most extensively studied contexts. Increased oxidative burden contributes to endothelial dysfunction, vascular inflammation, lipid peroxidation, and thrombotic events, all of which are key events in the development of atherosclerosis and other cardiovascular complications [36,37]. In addition, oxidative stress can significantly affect circulating blood cells, particularly RBCs, which are continuously exposed to high oxygen concentrations and therefore represent a highly sensitive cellular model for investigating redox imbalance [38,39].

Given the pivotal role of oxidative stress in cardiovascular pathophysiology, the identification of effective preventive strategies represents a major challenge in biomedical research. In this context, dietary interventions have gained increasing relevance, particularly those involving foods rich in natural antioxidants. Epidemiological and experimental studies have consistently shown that diets abundant in fruits and vegetables are associated with a reduced risk of CVD, largely due to the presence of bioactive phytochemicals capable of counteracting oxidative stress and inflammation [40,41].

Among these foods, tomato (*Solanum lycopersicum*) represents one of the most widely consumed vegetables worldwide and has attracted considerable scientific interest because of its potential health-promoting properties [42]. Tomatoes are traditionally recognized as a major dietary source of lycopene, a carotenoid with strong antioxidant and anti-inflammatory activities. Lycopene has been shown to reduce oxidative damage, improve endothelial function, and modulate lipid metabolism, thereby contributing to cardiovascular protection [43,44]. However, the beneficial effects of tomatoes cannot be attributed solely to lycopene. In fact, tomatoes also contain a complex mixture of bioactive compounds, including vitamins, minerals, and a wide range of polyphenols, such as flavonoids and phenolic acids, which may act synergistically to enhance their antioxidant potential [45]. Furthermore, it should be considered that tomatoes represent a complex food matrix in which multiple bioactive compounds coexist and may interact synergistically. Although the present study focuses on polyphenol-enriched extracts, other constituents naturally present in tomatoes, such as vitamins and carotenoids, could contribute to the overall biological effects. In addition, under real dietary conditions, tomatoes are rarely consumed alone but are commonly processed and combined with other ingredients, such as oils, which can enhance the bioavailability and activity of lipophilic and hydrophilic compounds [46]. This suggests that synergistic interactions may occur not only among tomato constituents but also between tomatoes and other components of the diet, potentially amplifying their protective effects in vivo.

It should be noted that the extraction method employed in this study (hydroalcoholic solvent) is expected to preferentially recover hydrophilic compounds, such as polyphenols, while limiting the extraction of lipophilic carotenoids. However, since carotenoid content was not directly quantified, their potential contribution, although likely minimal, cannot be completely excluded.

In the present study, we investigated the protective effects of polyphenolic extracts obtained from two widely consumed tomato cultivars, Piccadilly and Piennolo, on human RBCs exposed to Hg-induced oxidative stress. The first important observation emerging from our results concerns the chemical characterization of the extracts. In particular, the Piennolo cultivar exhibited significantly higher levels of total polyphenols, flavonoids, and ortho-diphenols compared with Piccadilly tomatoes. These findings are consistent with previous studies reporting that traditional or geographically specific cultivars may accumulate higher levels of bioactive phytochemicals, likely due to genetic and environmental factors influencing secondary metabolism [47]. Despite the quantitative differences observed in phenolic composition, both extracts showed comparable antioxidant capacity in the TAC and FRAP assays.

The data presented in this study demonstrate that polyphenol-rich fractions derived from two tomato cultivars exert a significant protective effect against Hg-induced oxidative damage in human RBCs. These findings are consistent with an increasing body of literature highlighting the ability of dietary polyphenols to counteract heavy metal-induced oxidative stress and preserve RBC functionality [24]. Previous studies using polyphenol-rich extracts from foods such as olive oil or apple have similarly reported protective effects in Hg-exposed RBCs, including the reduction of ROS production, restoration of intracellular antioxidant systems, and preservation of membrane integrity [10].

The biochemical alterations induced by HgCl_2_ observed in our experimental model are in line with the well-characterized mechanisms of Hg toxicity. As described in the introduction, Hg has a strong affinity for SH groups and therefore interacts with cysteine residues present in proteins and low-molecular-weight thiols, such as GSH. This interaction leads to inhibition of antioxidant enzymes, depletion of intracellular thiols, and disruption of redox homeostasis [48,49]. In agreement with these mechanisms, our results show that Hg exposure causes a marked depletion of intracellular SH groups and GSH levels, accompanied by increased ROS production, lipid peroxidation, and hemoglobin oxidation.

The ability of tomato extracts to prevent the depletion of intracellular SH groups suggests that tomato polyphenols may contribute to cellular redox balance through multiple mechanisms. First, polyphenols are well known to directly scavenge ROS, thereby reducing oxidative damage to cellular components [50]. Second, several phenolic compounds possess metal-chelating properties that may limit the redox activity of transition metals and prevent the propagation of oxidative reactions [51,52]. Third, polyphenols may contribute to the preservation of endogenous antioxidant systems by protecting GSH from oxidative consumption or by stabilizing thiol-containing proteins [53]. These mechanisms could explain the significant restoration of intracellular GSH levels observed in extract-treated cells, particularly at the highest concentration tested.

The reduction in MetHb formation observed in our study further supports the protective role of tomato polyphenols on RBC redox homeostasis. MetHb formation is a direct consequence of hemoglobin oxidation, which occurs under conditions of excessive ROS production. Previous studies have shown that antioxidant polyphenols can protect hemoglobin from oxidative conversion by stabilizing the ferrous state of the heme iron and by limiting ROS-mediated oxidative reactions [54]. In this context, the observed decrease in MetHb levels in extract-treated cells indicates that tomato polyphenols are able to preserve the functional integrity of hemoglobin and therefore the oxygen-carrying capacity of RBCs.

Another important aspect emerging from our results concerns the attenuation of lipid peroxidation, as demonstrated by the reduction of TBARS levels in RBCs treated with tomato extracts. The RBC membrane is particularly susceptible to lipid peroxidation due to its high content of polyunsaturated fatty acids and continuous exposure to oxygen. Oxidative damage to membrane lipids can lead to alterations in membrane fluidity, increased rigidity, and impaired deformability of RBCs [55]. Polyphenols are known to interact with lipid bilayers and stabilize membrane structure, thereby limiting lipid peroxidation and preserving membrane functionality [56]. This membrane-stabilizing effect may therefore represent an additional mechanism underlying the protective effects observed in our study.

The analysis of ROS production kinetics provided further insights into the antioxidant activity of tomato extracts. As expected, Hg exposure induced a rapid and time-dependent increase in intracellular ROS levels. Pretreatment with tomato extracts significantly attenuated this increase in a concentration-dependent manner, with the highest concentration restoring ROS levels close to those observed in untreated cells. These findings are consistent with previous studies demonstrating that plant-derived polyphenols can effectively modulate intracellular ROS generation in RBCs exposed to oxidative stressors.

Although both tomato cultivars showed similar protective effects in most assays, Pien. extracts displayed a slightly greater ability to reduce ROS levels at higher concentrations. This observation may be related to the higher content of polyphenols, flavonoids, and ortho-diphenols detected in this cultivar. Phenolic compounds such as flavonoids and phenolic acids have been extensively reported to possess strong radical-scavenging and membrane-protective properties, which may contribute to the enhanced antioxidant activity observed [57,58].

These findings are further supported by the HPLC analysis, which revealed a markedly different phenolic profile between the two cultivars. In particular, Pien. extracts showed significantly higher concentrations of *p*-coumaric acid and rutin compared to Piccadilly, while chlorogenic acid was not detected. Conversely, Picc. samples contained all three compounds, albeit at substantially lower levels. The higher abundance of specific phenolic compounds in Pien. may contribute to its slightly stronger antioxidant performance, especially at higher concentrations.

Rutin, a well-known flavonoid, has been widely reported to exert potent antioxidant and membrane-stabilizing effects in erythrocytes, including the ability to scavenge ROS and protect hemoglobin from oxidative damage [59]. Similarly, p-coumaric acid has been associated with radical-scavenging activity and the modulation of redox-sensitive pathways [60,61]. Therefore, the higher levels of these compounds in Pien. extracts may partially explain the enhanced protective effects observed in this cultivar.

In contrast, the presence of chlorogenic acid in Picc. extracts, although at low concentrations, suggests that different combinations of phenolic compounds may contribute to the overall antioxidant response through synergistic mechanisms. This supports the concept that not only the total phenolic content, but also the qualitative composition of polyphenols, plays a crucial role in determining the biological activity of plant-derived extracts.

Among the various parameters analyzed in this study, PS externalization represents one of the most pathophysiologically relevant endpoints. Loss of phospholipid asymmetry in RBC membranes is a hallmark of eryptosis and is associated with increased procoagulant activity [62]. In particular, PS exposure on the outer leaflet of the RBC membrane promotes adhesion to endothelial cells and platelets and can contribute to thrombus formation [17]. Several studies have shown that exposure to Hg induces PS externalization by disrupting membrane phospholipid transport systems and by promoting oxidative stress-mediated signaling pathways [63,64].

The results obtained in our study clearly show that tomato extracts significantly reduce Hg-induced PS externalization in RBCs. From a mechanistic perspective, recent studies have demonstrated that oxidative stress can alter the activity of phospholipid translocases responsible for maintaining membrane asymmetry, particularly the ATP-dependent flippase ATP11C and the calcium-dependent scramblase PLSCR1 [15]. Hg exposure has been shown to inhibit flippase activity while simultaneously activating scramblase-mediated phospholipid scrambling, ultimately leading to PS exposure on the cell surface.

Interestingly, polyphenols have been reported to restore the physiological activity of these membrane enzymes by preserving intracellular ATP levels and glutathione content and by preventing oxidative modifications of membrane proteins [15,19]. Therefore, it is plausible that the reduction in PS exposure observed in our study may result not only from the direct antioxidant activity of tomato polyphenols but also from their ability to preserve the function of phospholipid translocases involved in membrane asymmetry maintenance.

Importantly, the results obtained in this study further support the concept that the health benefits associated with tomato consumption extend beyond the well-known effects of lycopene. While lycopene has long been considered the main bioactive compound responsible for the cardioprotective properties of tomatoes, increasing evidence indicates that other phytochemicals, particularly polyphenols, may play an equally relevant role. These compounds can act through complementary mechanisms, including antioxidant activity, metal chelation, and stabilization of cellular membranes, thereby contributing to the overall protective effects of tomato-derived bioactive molecules.

From a broader perspective, these findings highlight the potential relevance of tomato polyphenols as nutraceutical compounds in preventive strategies aimed at reducing oxidative stress and its associated cardiovascular risks. Considering the growing exposure to environmental pollutants such as heavy metals, dietary approaches based on the consumption of polyphenol-rich foods may represent an accessible and sustainable strategy to support cardiovascular health.

In conclusion, our results demonstrate that polyphenolic extracts from two tomato cultivars exert significant protective effects against Hg-induced oxidative damage in human RBCs, preserving redox balance, membrane integrity, and phospholipid asymmetry. These findings provide further evidence supporting the role of tomato-derived polyphenols as potential protective agents against oxidative stress-related cardiovascular alterations and reinforce the importance of considering the entire phytochemical profile of foods, rather than focusing solely on individual compounds such as lycopene, when evaluating their health-promoting properties. It is important to note that, although the biological effects observed in this study are likely related to the high polyphenolic content of the extracts; however, the contribution of other co-extracted bioactive compounds cannot be excluded.

## Figures and Tables

**Table 1:** Polyphenol, flavonoid and *ortho*-diphenol contents in two different tomato cultivars. Statistical analysis was performed using one-way ANOVA followed by Tukey’s test. *** (*p* < 0.001), ** (*p* < 0.01), * (*p* < 0.05) and ns (*p* > 0.05).

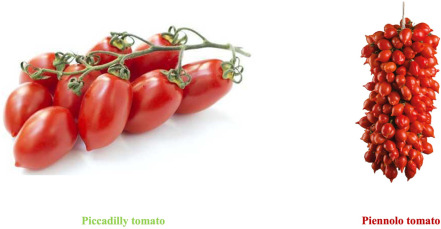			
Tomato Sample	Total Polyphenol Content (CAE mg Eq/100 g of Sample)	Total Flavonoid Content (Quercetin mg Eq/100 g of Sample)	Total *Ortho*-Diphenol Content (CAE mg Eq/100 g of Sample)
Piccadilly Tomato	42.56 mg/100 g	7.2 mg/100 g	6.56 mg/100 g
Piennolo Tomato	56.45 mg/100 g **	22.4 mg/100 g ***	9.85 mg/100 g *

## Data Availability

The original contributions presented in this study are included in the article. Further inquiries can be directed to the corresponding authors.

## Abbreviations

The following abbreviations are used in this manuscript:

CVD	Cardiovascular disease
RBC	Erythrocytes
FRAP	Ferric reducing antioxidant power
GSH	Glutathione
Hg	Mercury
MetHb	Methemoglobin
ROS	Reactive oxygen species
SH	Sulfhydryl group
TAC	Total antioxidant capacity
TBARS	Thiobarbituric acid reactive substances
